# Les cellulites orbitaires: étude prospective à propos de 75 cas

**DOI:** 10.11604/pamj.2015.22.340.7279

**Published:** 2015-12-10

**Authors:** Sarah Belghmaidi, Btissam Belhoucha, Ibtissam Hajji, Khaoula Hssaine, Youssef Rochdi, Hassan Nouri, Lahcen Aderdour, Abdelaziz Raji, Abdeljalil Moutaouakil

**Affiliations:** 1 Service d'Ophtalmologie, CHU Mohammed VI, Marrakech, Maroc; 2 Service d'Orl et Chirurgie Cervico-Faciale, CHU Mohammed VI, Marrakech, Maroc

**Keywords:** Cellulite preseptale, cellulite retroseptale, cecité, sinusite, preseptal cellulitis, retroseptal cellulitis, blindness, sinusitis

## Abstract

Les cellulites orbitaires est une affection grave par ses complications aussi bien locales, locorégionales que générales, pouvant engager le pronostic vital et fonctionnel, surtout lorsque le diagnostic est tardif et la prise en charge inappropriée. Le but de cette étude est de décrire les aspects épidémiologiques, cliniques, thérapeutiques et évolutifs des cellulites orbitaires et d'insister sur la nécessité d'un diagnostic et d'un traitement précoces, afin d’éviter ses complications. Il s'agit d'une étude prospective concernent 75 patients présentant une cellulite orbitaire, menée au service d'Ophtalmologie et d'ORL au CHU Mohammed VI de Marrakech, de Septembre 2010 au Avril 2014. L’âge moyen des patients était de 24 ans allant de 2 ans à 70 ans. La porte d'entrée était dominée par l'atteinte sinusienne retrouvée chez 43 malades. L'examen ophtalmologique a montré une BAV chez 20% des patients avec une cécité bilatérale chez un patient et unilatérale chez 3, un chémosis (82%), une exophtalmie (85,71%), un ptosis (30%), une ophtalmoplégie (66%), une fistule orbitaire (4 cas), et une kératite d'exposition chez 8 cas. L'analyse des résultats tomodensitométriques a noté: 24 cas de cellulite pré septale (45%), 20 cas de cellulite orbitaire (15%), 2 cas d'abcès sous périosté (5%) et 14 cas d'abcès orbitaire (35%). 20 patients ont bénéficié d'un traitement chirurgical associé au traitement médical, ayant consisté en un drainage de l'abcès orbitaire dans 24 cas, une ethmoidectomie antérieure par voie endoscopique avec drainage d'un abcès sous-périosté dans 2 cas à et un drainage d'une collection abcédée des parties molles dans 6 cas. La cellulite orbitaire est une urgence thérapeutique qui met en jeu le pronostic visuel et vital. Causés le plus fréquemment par les traumatismes oculaires post chirurgicale ou AVP, les sinusites, les fractures orbitaires, et les corps étrangers intraoculaires. Les infections rétro-septales sont les plus graves, nécessitant une exploration par imagerie en coupes. L’évolution de la cellulite orbitaire est toujours grave en l'absence d'un traitement médical et chirurgical strict. Le traitement précoce et adapté représente un élément pronostique très important. Les cellulites orbitaires est une affection grave pouvant engager le pronostic vital et fonctionnel, surtout lorsque le diagnostic est tardif et la prise en charge inappropriée.

## Introduction

Les cellulites orbitaires sont définies par la présence d'une tuméfaction orbitaire aiguë inflammatoire d'origine infectieuse. Elles représentent la pathologie orbitaire primitive la plus fréquente. La sinusite est l’étiologie la plus fréquente. C'est une pathologie grave par ses complications aussi bien locales, locorégionales que générales. On distingue les cellulites périorbitaires ou préseptales, situées en avant du septum orbitaire et d’évolution le plus souvent favorable, et les cellulites rétroseptales, plus rares et pouvant mettre en jeu le pronostic vital ou fonctionnel. Le traitement est avant tout médical et la chirurgie n'est nécessaire qu'en cas d'abcédation [1, 2]. Le but de cette étude est de décrire les aspects épidémiologiques, cliniques, thérapeutiques et évolutifs des cellulites orbitaires et d'insister sur la nécessité d'un diagnostic et d'un traitement précoces, afin d’éviter ses complications.

## Méthodes

Il s'agit d'une étude prospective concernent 75 patients, menée au service d'Ophtalmologie et d'ORL au CHU Mohammed VI de Marrakech, de Septembre 2010 au Avril 2014. Tous les malades ont eu une consultation ophtalmologique et ORL. Sur chaque dossier, nous avons relevé l’état civil du patient, le délai de consultation, le traitement avant l'admission et les données de l'examen clinique initial. L'examen ophtalmologique comprenait la mesure l'acuité visuelle. La présence d'une exophtalmie, d'une lagophtalmie, d'un chémosis ou d'une ophtalmoplégie était systématiquement notée. L’état du segment antérieur (transparence et intégrité de la cornée) et du segment postérieur a été secondairement apprécié. L'examen ORL comprenait La rhinoscopie antérieur, l'examen du palais, des gencives et la palpation des sinus, et la rhinocavoscopie. Des examens d'imageries médicales (tomodensitométrie), des examens de pus prélevé permettaient de procéder au bilan étiologique ou d'extension et d'isoler les germes en cause. Nous avons relevé le type (médical et/ou chirurgical) et la nature du traitement dont le patient a bénéficié, la voie d'administration, l’évolution sous traitement. La classification anatomo-clinique de Chandler était utilisée pour classer les malades en fonction de la localisation de l'infection.

## Résultats

**Figure 1:**
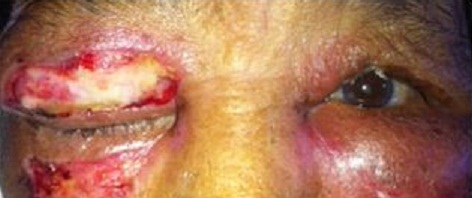
Cellulite préseptale bilatérale compliquant un traumatisme de la face

**Figure 2:**
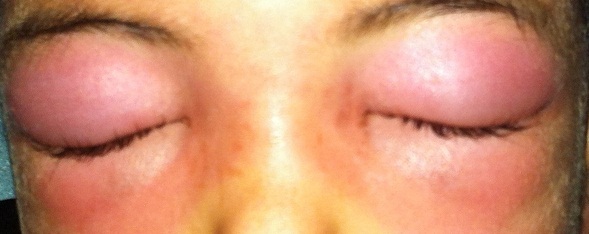
Aspect de cellulite préseptale complicant une ethmodite chez un enfant de 08 ans

**Figure 3:**
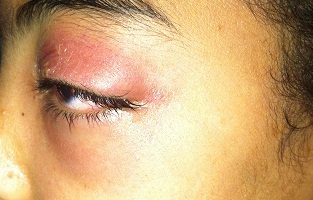
Aspect de cellulite rétroseptale au stade d'abcès sous périosté

**Figure 4:**
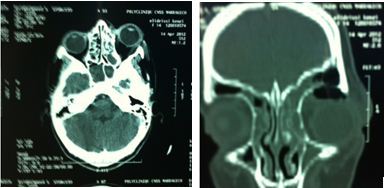
TDM orbitaire en coupe axiale et coronale objectivant un abcès orbitaire compliquant une sinusite

**Figure 5:**
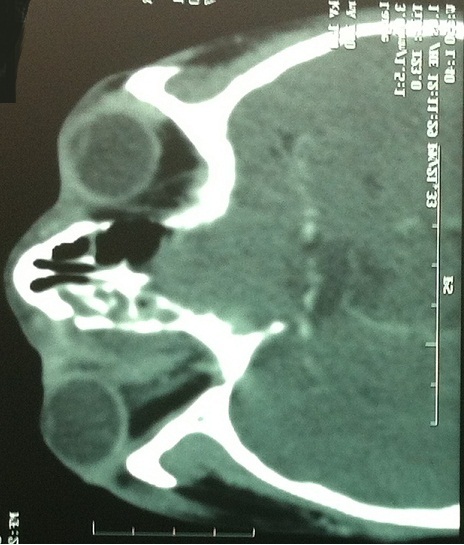
TDM orbitaire en coupe axiale montrant un abcès sous périosté

Sur les 75 patients atteints de cellulite de la région orbitaire, vingt trois (65,7%) avaient une cellulite rétroseptale et douze (32,28%) avaient une cellulite préseptale. On a noté une prédominance masculine avec un sex ratio de 2,13. Le retard diagnostique était de 7 jours en moyenne dans les cellulites rétroseptales et 2 jours seulement dans les localisations préseptales. L’âge moyen des patients était de 24 ans allant de 2 ans à 70 ans (Tableau 1), (Figure 1): l’œdème palpébral et la douleur périorbitaire étaient constants, ces symptômes étaient associés à une fièvre entre 38,5 et 40 C chez 80% du patient sans signes neuro méningés associés (Tableau 2) (Figure 2 et Figure 3). La rhinocavoscopie a objectivé une muqueuse inflammatoire dans 45% du cas, des sécrétions purulentes au niveau du méat moyen dans 25% du cas associé à un mouvais état buccodentaire dans 55% du cas (Tableau 3) (Figure 4 et Figure 5). Les prélèvements bactériologiques ont été réalisés chez 6 cas d'abcès orbitaire, ils ont révélé une flore poly microbienne dans 04 cas, le troisième est revenu négatif. En cas de cellulite préseptale non collectée, la prise en charge a été faite en ambulatoire. Le traitement par l'association amoxicilline-acideclavulanique par voie orale a été proposé avec contrôle clinique du patient à 24-48 heures. Tous nos patients présentant une cellulite rétro septale ont été hospitalisés et ont reçu un traitement médical à base d'antibiothérapie par voie systémique pendant 10 jours en moyenne (à base d'amoxicilineacide clavulanique 100 mg/Kg/j + aminoside + imidazolé chez 36 patients, et à base de céphalosporine 3ème génération + aminoside + imidazolé chez 10.) suivi d'un relais par voie orale pendant 2 à 3 semaines. 20 patients ont bénéficié d'un traitement chirurgical, ayant consisté en un drainage de l'abcès orbitaire dans 24 cas, une ethmoidectomie antérieure par voie endoscopique avec drainage d'un abcès sous-périosté dans 2 cas à et un drainage d'une collection abcédée des parties molles dans 6 cas (4 cas d'abcès palpébral et 3 cas d'abcès frontal compliquant une cellulite preseptale) et une tarsorraphie chez 2 patients. L’évolution a été marquée par la persistance d'une exophtalmie avec paralysie du III chez un patient, et la persistance d'une opacité coréenne chez 4 autres patients, une TDM de contrôle a révélé un abcès sous-périosté qui a été drainé ultérieurement par voie externe. Aucun cas de récidive ni mortalité n'a été noté.

**Tableau 1:** La porte d'entrée de la cellulite orbitaire

La porte d'entrée	Le nombre de malades
l'atteinte sinusienne	43 malades
une pyodermite	8 malades
une plaie infectée post traumatique	16 malades
aucune porte d'entrée n'est mise en évidence	8 malades

**Tableau 2:** Les résultats de l'examen ophtalmologique

L'examen ophtalmologique	Pourcentage
BAV	20%
une cécité bilatérale	1, 33%
unilatérale	4%
un chémosis	82%
une exophtalmie	85,71%
un ptosis	30%
une ophtalmoplégie	66%
une fistule orbitaire	5,33%
une kératite d'exposition	10,66%
une mydriase	1,33%
un œdème papillaire	8%
Une dacryocystite	13, 33%

**Tableau 3:** L'analyse des résultats tomodensitométriques de la cellulite orbitaire

L'analyse des résultats tomodensitométriques	Le nombre de malades
cellulite pré septale	24 cas
cellulite orbitaire	20 cas
d'abcès sous périosté	2 cas
d'abcès orbitaire	14 cas
une pansinusite	8 cas
une sinusite maxillaire	4 cas
une sinusite éthmoido frontale	16 cas

## Discussion

Diagnostiquée et traitée précocement, la cellulite orbitaire évolue bien et sans séquelles; tout retard diagnostique et ou thérapeutique peut être source de complications graves pouvant engager le pronostic fonctionnel et même vital. Le spécialiste devra procéder au diagnostic rapide, à l’évaluation du retentissement oculaire, sur la motilité et la vision et à la mise en route du traitement médical. La chirurgie restera réservée aux complications. Dans notre étude, les cellulites rétro septales avaient une proportion plus élevée que dans les localisations pré septales, cette fréquence peut être expliquée par le retard diagnostique plus élevé. L'origine sinusienne est impliquée dans au moins deux tiers des cellulites orbitaires de l'adulte et dans 90% des cellulites de l'enfant [1, 2]. La seconde cause décrite dans la littérature est cutanée, faite essentiellement d'infections cutanées et des traumatismes avec plaie surinfectée [2–4]. Les signes cliniques dépendent de la localisation de l'infection objectivée par la classification anatomo-clinique de Chandler [5]. La forme rétro septale reste une cause possible de cécité, voire de mortalité en cas de complication(s). Dans notre série on a noté une cécité bilatérale chez un cas et unilatérale chez 2. Aucune thrombose de sinus caverneux n'a été objectivée dans notre étude. Sur une petite série de 23 patients, Hodges et al. Rapportent 52% de cécité et 4% de mortalité par thrombose du sinus caverneux [6]. La cécité est secondaire à une neuropathie optique mécanique, à une origine vasculaire par ischémie, une thrombophlébite ou une origine inflammatoire (neurite infectieuse) [7]. La thrombose de la loge caverneuse était une pathologie fréquente avant l'avènement de l'antibiothérapie avec une mortalité de 100% [8], avec les traitements antibiotiques ce taux a diminué pour atteindre actuellement des pourcentages entre 23 et 50% [9]. Sur le plan bactériologique, le germe dépend de la localisation, de la porte d'entrée et de l’âge du patient [10]. La prise d'antibiothérapie préalable dans 30% des cas expliquerait probablement le nombre faible de prélèvements positifs dans notre série. La tomodensitométrie permet le diagnostic des cellulites rétro septales avec un bilan précis des lésions. Le traitement médical est basé sur l'antibiothérapie parentérale à large spectre: amoxicilline - acide clavulanique ou céfotaxime associé à un aminoside en fonction de l'orientation clinique. Cette antibiothérapie constitue le traitement de première intention devant les cas de cellulites pré septales, de cellulites orbitaires (stade 2) ou d'abcès sous-périosté (stade 3) [1]. Par contre, le traitement des abcès orbitaires (stade 4) est plus controversé [11]. Si le traitement chirurgical qui est actuellement le plus souvent sous forme d'un drainage per-endoscopique des abcès orbitaires [12], est formel pour certains, pour d'autres, un traitement antibiotique intraveineux associé à une surveillance échographique suffirait le plus souvent [13].

## Conclusion

Les cellulites orbitaires sont des affections graves pouvant engager le pronostic vital et fonctionnel, surtout lorsque le diagnostic est tardif et la prise en charge inappropriée.
